# Cytokine profiling in the sub-silicone oil fluid after vitrectomy surgeries for refractory retinal diseases

**DOI:** 10.1038/s41598-017-03124-x

**Published:** 2017-05-25

**Authors:** Hiroki Kaneko, Kei Takayama, Tetsu Asami, Yasuki Ito, Taichi Tsunekawa, Takeshi Iwase, Yasuhito Funahashi, Shinji Ueno, Norie Nonobe, Shunsuke Yasuda, Ayana Suzumura, Hideyuki Shimizu, Reona Kimoto, Shiang-Jyi Hwang, Hiroko Terasaki

**Affiliations:** 1 Department of Ophthalmology, Nagoya University Graduate School of Medicine, Nagoya, Japan; 2 Miyake Eye Hospital, Nagoya, Japan; 3 Department of Urology, Nagoya University Graduate School of Medicine, Nagoya, Japan; 4 Laboratory of Bell Research Center–Department of Obstetrics and Gynecology collaborative research, Nagoya University Graduate School of Medicine, Nagoya, Japan

## Abstract

Silicone oil (SO) is an intraocular surgical adjuvant that reduces the surgical complications in refractory retinal diseases, although membrane and cellular proliferation is often seen even in SO-filled eyes. We hypothesised that the fluid in the space between the SO and the retina, named the “sub-silicone oil fluid (SOF)”, enhances these biological responses. We proposed a safe method for SOF extraction. We also analysed inflammatory cytokine expressions and SOF osmotic pressures from eyes with rhegmatogenous retinal detachment (RRD), proliferative diabetic retinopathy (PDR), proliferative vitreoretinopathy (PVR) and macular hole-associated retinal detachment (MHRD). Interleukin (IL)-10, IL-12p40, IL-6, monocyte chemotactic protein-1, and vascular endothelial growth factor (VEGF) in the SOF with PVR were significantly higher than in those with RRD or MHRD. Fibroblast growth factor-2, IL-10, IL-12p40, IL-8, VEGF, and transforming growth factor beta 1 levels in eyes with exacerbated PDR indicated a significantly higher expression than those with simple PDR. IL-6 and tumour necrosis factor alpha in eyes with exacerbated PVR demonstrated a significantly higher expression than in those with simple PVR. However, there was no difference in SOF osmotic pressure between group of each disease. These studies indicate that disease-specific SOF is a significant reflection of disease status.

## Introduction

Among the robust improvements in eye surgeries, vitrectomy surgery has greatly contributed to the successful treatment of severe retinal diseases, e.g., rhegmatogenous retinal detachment (RRD), proliferative diabetic retinopathy (PDR) and proliferative vitreoretinopathy (PVR). Over the last four decades since the concept of vitrectomy surgeries was first reported^1^, improvement to surgical instruments as well as surgical adjuvant therapy has contributed to raise the surgical success rate associated with these retinal diseases. Silicone oil (SO) was first used as a surgical adjuvant for retinal surgery in the 1960 s^2^ and since then large clinical studies have been performed to understand the risks and benefits of its use for retinal surgeries^3–9^. It has been reported that SO caused glaucoma and unexplained vision loss after SO removal^10–12^. In addition, sustained SO in the eye caused undesired complications such as intraretinal SO residue^13^ and migration of SO into the ventricles of the brain^14^. Therefore, understanding biological changes in SO-filled eyes is critical for the precise application of SO during surgery.

In a regular case with vitrectomy surgery that requires SO tamponade, SO is evacuated after the retina is attached or at the quiescent condition, usually several weeks to months after the primary surgery. Revision surgery is also required before the eyes reach a quiescent condition. In such cases, membrane and cellular proliferation occur even under an SO tamponade. During SO removal, there is certain amount of fluid in the space between SO and the retina in the eye. In 2004, Asaria *et al*.^15^ named the fluid “retro-oil fluid” and they studied cytokine levels from 13 eyes with PVR. While their idea was very innovative, their sample size was small and only three cytokine parameters were reviewed: fibroblast growth factor-2 (FGF-2), interleukin (IL)-6 and transforming growth factor-β2 (TGFβ2). In the more than ten years since their first report, better and safer surgical instruments have been developed to extract the fluid under SO. In addition, advanced biological assays enable us to measure multiple cytokines from a tiny sample amount. In this study, we newly named the fluid under SO as “sub-silicone oil fluid (SOF)” and propose a safe method to extract SOF using currently available devices. Moreover, we examined multiple inflammatory cytokine levels and osmotic pressures not only of SOF but also of original vitreous fluid from the same eyes with representative severe retinal diseases including RD, PVR, PDR, and macular hole-associated retinal detachment (MHRD).

## Results

### Patient characteristics

In total, 50 SOF samples were collected in this study from which 13 vitreous fluid samples were also collected during the primary vitrectomy surgery. The primary retinal diseases were RD, PDR, PVR, and MHRD and patient characteristics are listed in Table 1.

**Table 1:** Patients’ characteristics.

	No. of patients (male)	Age	Duration of SO tamponade (months)
RRD	15 (13)	55.4 ± 19.9	4.1 ± 2.1
PDR	17 (12)	47.0 ± 12.0	4.5 ± 2.0
PVR	14 (10)	50.6 ± 25.3	4.0 ± 2.1
MHRD	4 (1)	55.4 ± 19.9	7.0 ± 2.3

RRD: Rhegmatogenous retinal detachment, PDR: Proliferative diabetic retinopathy, PVR: Proliferative vitreoretinopathy, MHRD: Macular hole-associated retinal detachment.

### Extraction of SOF and vitreous fluid

**Figure 1:**
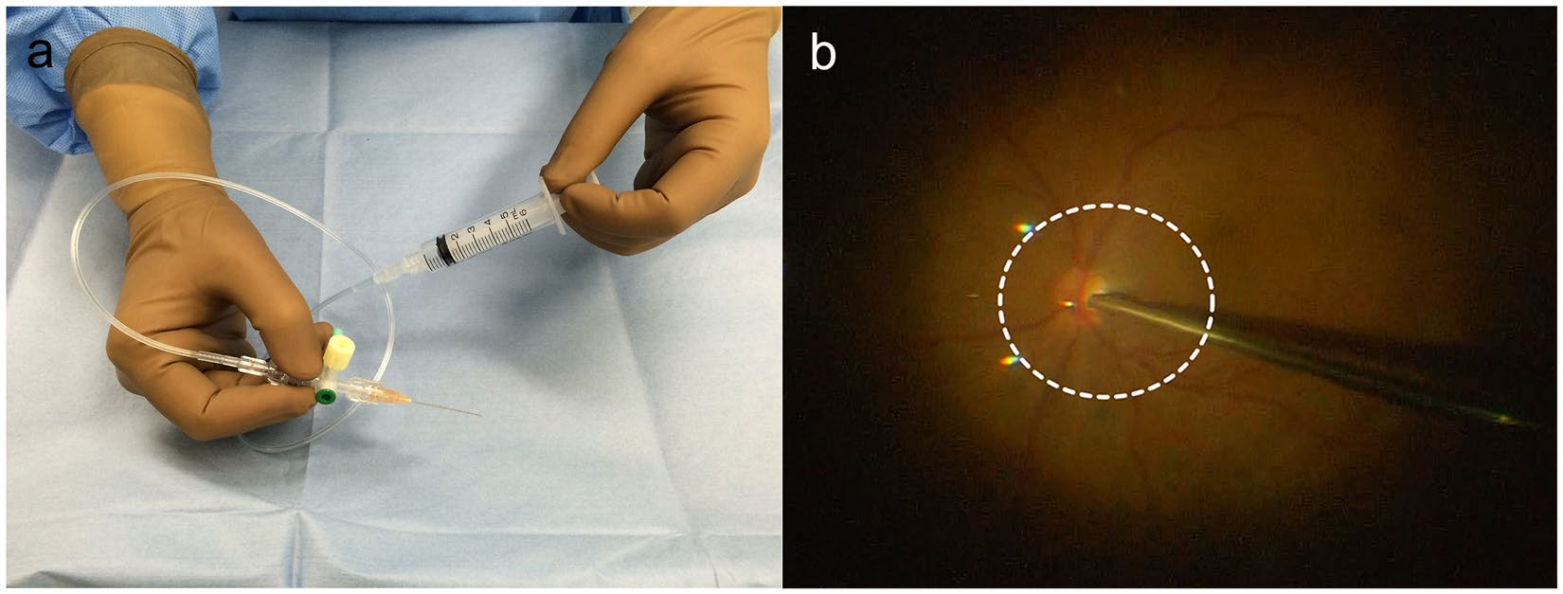
Method for sub-silicone oil fluid (SOF) extraction. **(a)** Setting up an SOF extraction kit. **(b)** Once the surgeon places the tip of the blunt needle above the optic nerve the assistant pulls back the plunger of the syringe. The white dotted line indicates the border between the fluid and silicone oil.

First, we established a safe and easy method to extract SOF. One of the greatest improvements in the recent surgical method is the advanced development of the surgical microscope: a wide-angle viewing system. In this study, we used Resight (Carl Zeiss Meditec AG, Jena, Germany) as it enables surgeons to clearly view the fundus even in SO-filled eyes. We prepared a 25 G blunt needle (30 mm length), 3-way stopcock, medical extension tube (inner diameter: 1.1 mm) and a 5-mL syringe to make the SOF extraction kit. Figure 1 shows the SOF extraction kit and the fundus image when SOF is extracted under a Resight microscope. Supplementary material 1 shows the surgical video under the surgical microscope as a surgical illumination reflexed on the SOF while it was extracted.

### Inflammatory Cytokines

**Figure 2:**
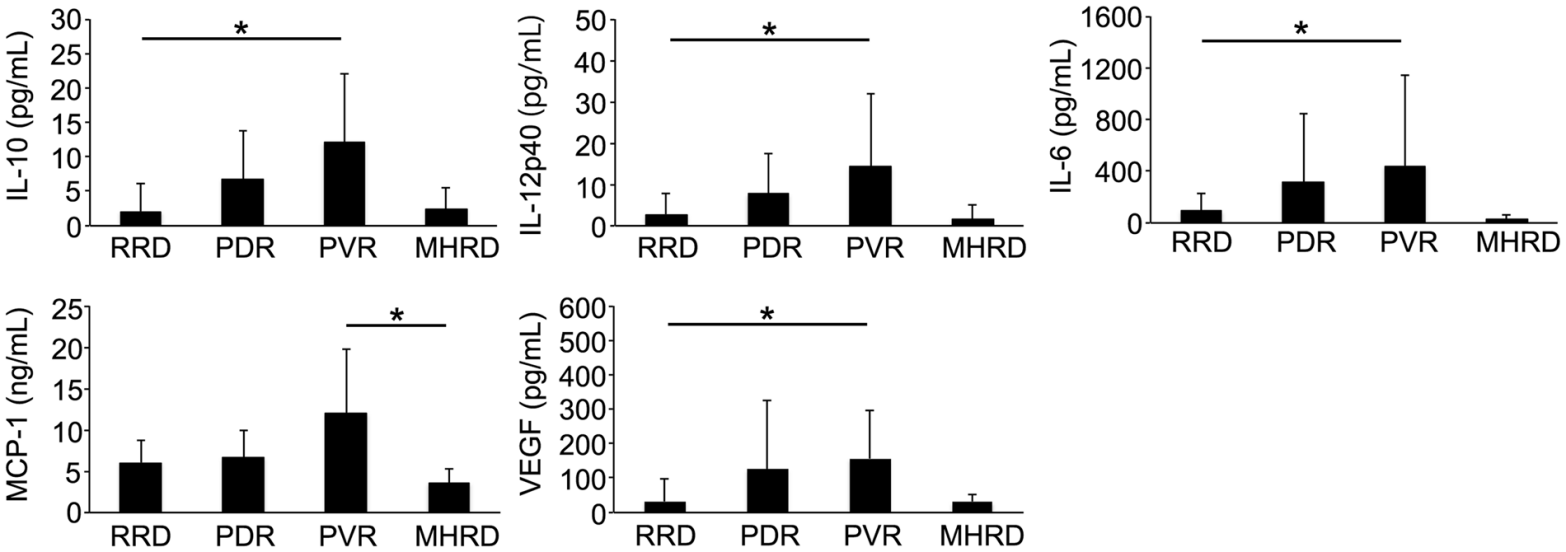
Comparison of cytokine levels of sub-silicone oil fluid (SOF) and disease. The IL-10, IL-12p40, IL-6 and VEGF in SOF from eyes in patients with proliferative vitreoretinopathy (PVR) were significantly higher than those with rhegmatogenous retinal detachment (RRD). The monocyte chemoattractive protein-1 (MCP-1) in the SOF from eyes with PVR was significantly higher than those with macular hole-associated retinal detachment (MHRD).

The major inflammatory cytokine levels analysed were FGF-2, interferon-γ (IFN-γ), IL-10, IL-12p40, IL-1β, IL-6, IL-8, monocyte chemotactic protein-1 (MCP-1), tumour necrosis factor-α (TNFα), vascular endothelial growth factor (VEGF) and transforming growth factor-β1 (TGFβ1). These cytokine levels in the SOF for each disease are listed in Table 2. Compared with RRD, the SOF from eyes with PVR showed a higher expression in IL-10 (5.9-fold, P = 0.008), IL-12p40 (5.1-fold, P = 0,043), IL-6 (4.6-fold, P = 0.009), and VEGF (4.9-fold, P = 0.040). Compared with MHRD, the SOF from eyes with PVR showed a higher MCP-1 expression (3.4-fold, P = 0.023) (Fig. 2). The amount of SOF was 312.1 ± 326.7 μL. The results of correlation between the amount of SOF and cytokine levels are listed in Table 3. Although *P* values from IFNγ and TNFα were < 0.05, all *r* values were between −0.3 and 0.3, indicating that the cytokine levels were independent of the amount of SOF.

**Table 2:** Cytokine levels in SOF.

Number of Samples		RRD	PDR	PVR	MHRD
		15	17	14	4
FGF-2	(pg/mL)	26.1 ± 22.9	37.4 ± 54.4	35.4 ± 30.5	11.5 ± 10.8
IFNγ	(pg/mL)	0 ± 0	2.3 ± 9.6	1.2 ± 3.0	0 ± 0
IL-10	(pg/mL)	2.1 ± 4.0	6.8 ± 7.1	12.2 ± 9.9	2.4 ± 3.2
IL-12p40	(pg/mL)	2.8 ± 5.0	7.7 ± 9.8	14.2 ± 17.6	1.7 ± 3.3
IL-1β	(pg/mL)	0 ± 0	1.5 ± 4.4	0 ± 0	0 ± 0
IL-6	(pg/mL)	95.3 ± 133.6	317.9 ± 524.5	440.2 ± 701.4	31.7 ± 34.8
IL-8	(pg/mL)	78.1 ± 65.1	79.4 ± 97.5	82.7 ± 40.7	30.9 ± 15.5
MCP-1	(pg/mL)	6044.6 ± 2734.6	6670.5 ± 3251.1	12042.8 ± 7795.1	3582.5 ± 1764.2
TNFα	(pg/mL)	0.8 ± 2.2	0.9 ± 2.1	1.0 ± 2.5	1.3 ± 1.7
VEGF	(pg/mL)	32.0 ± 64.9	126.3 ± 197.3	156.6 ± 139.8	29.7 ± 23.6
TGFβ1	(pg/mL)	105.7 ± 82.8	143.2 ± 125.4	215.6 ± 123.4	83.1 ± 31.2

RRD: Rhegtamogenous retinal detachment, PDR: Proliferative diabetic retinopathy, PVR: Proliferative vitreoretinopathy, MHRD: Macular hole-associated retinal detachment.

**Table 3:** Correlation between the amount of SOF and cytokine levels.

	*r*	*p* value
FGF-2	−0.2004	0.1674
IFNγ	−0.2958	0.0391
IL-10	−0.0729	0.6184
IL-12p40	−0.2311	0.1101
IL-1β	−0.1301	0.3730
IL-6	−0.1635	0.2616
IL-8	0.2562	0.0756
MCP-1	−0.0771	0.5987
TNFα	0.2933	0.0408
VEGF	−0.2580	0.0735
TGFβ1	0.0580	0.7020

**Figure 3:**
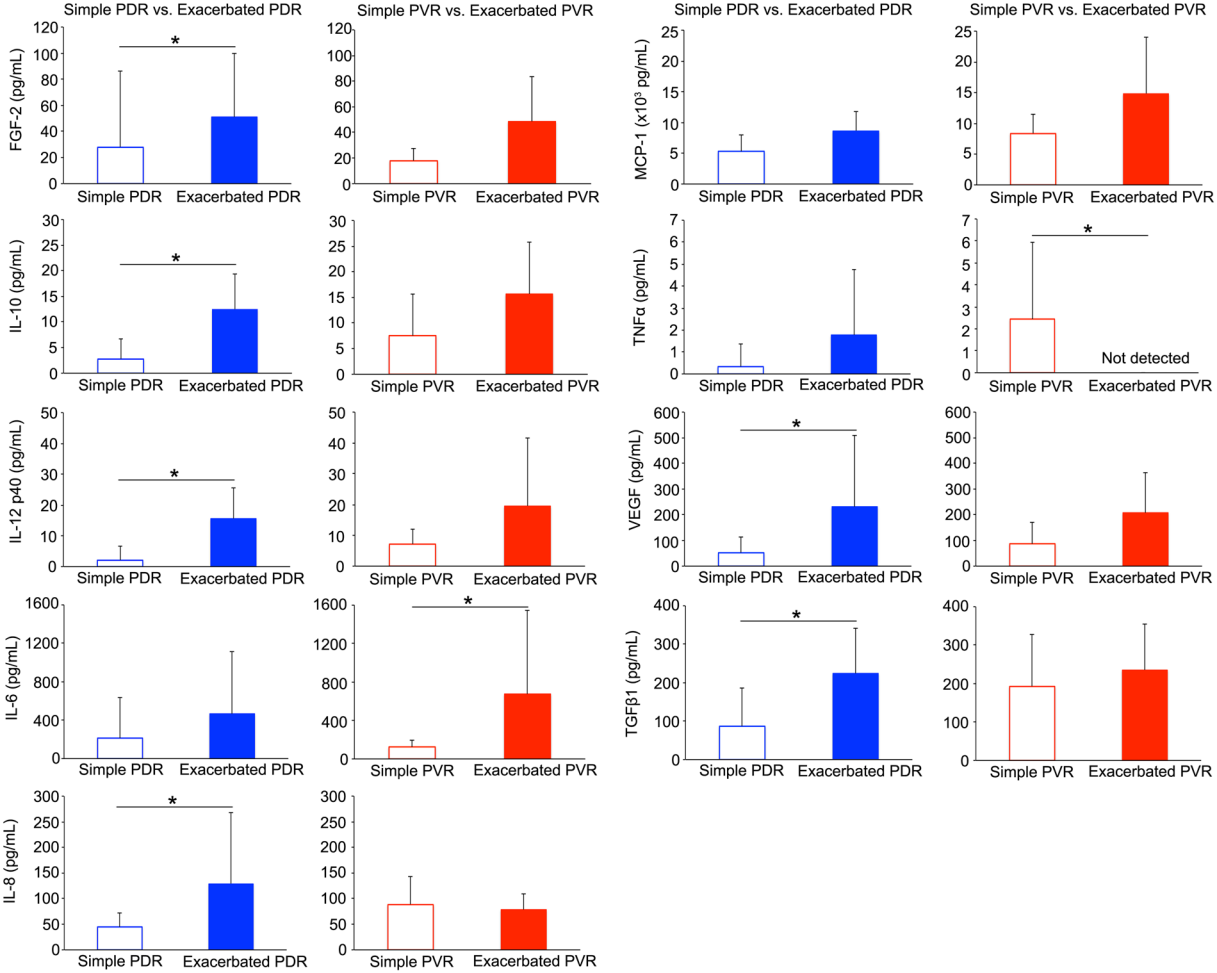
Difference in cytokine levels dependent on disease severity. The FGF-2, IL-10, IL-12p40, IL-8, VEGF and TGFβ1 levels in eyes with exacerbated PDR showed a significantly higher expression than those with simple PDR. The IL-6 and TNFα in eyes with exacerbated PVR showed significantly higher expression than those with simple PVR.

We further focused on differences in cytokine levels from eyes with the same disease but with a different clinical course. Depending on whether the eyes had a simple SO evacuation or required an additional surgical intervention, PDR and PVR were divided into a simple or exacerbated group, respectively. The differences among the surgical procedures are listed in Table 4. Although there were significant differences in the duration of second surgeries between simple and exacerbated PDR as well as between simple and exacerbated PVR, there was no difference in the duration of first surgeries and the number of endolaser photocoagulation, indicating that increased cytokine levels in SOF were not simply because of the difference in the surgical procedures during the primary vitrectomy. The average cytokine levels in each group are listed in Table 5. The SOF from eyes with exacerbated PDR showed a higher expression in FGF-2 (1.8-fold, P = 0.018), IL-10 (4.5-fold, P = 0.0014), IL-12p40 (7.4-fold, P = 0.002), IL-8 (2.9-fold, P = 0.014), VEGF (4.5-fold, P = 0.020) and TGFβ1 (2.6-fold, P = 0.014) compared with simple PDR. The SOF from eyes with exacerbated PVR showed a higher expression in IL-6 (5.4-fold, P = 0.003) and TNFα (none of the SOF samples in exacerbated PVR expressed TNFα, P = 0.003) compared with simple PVR. However, there was no significant difference in MCP-1 levels between simple PDR and exacerbated PDR or simple PVR and exacerbated PVR (Fig. 3).

**Table 4:** Surgical procedures of PDR and PVR.

		Time of surgery (minutes)		Endolaser photocoagulation (shots)	Membrane remove (+/−)	Encircling (+/−)	Retinotomy (+/−)
		1st surgery	2nd surgery				
PDR	Simple	92.8 ± 49.9	32.9 ± 10.6	1493.9 ± 935.0	10/0	0/10	0/10
	Exacerbated	101.3 ± 31.1	73.1 ± 34.2	1715.0 ± 517.9	7/0	2/5	1/6
	*P* value	0.46	0.0062	0.84			
PVR	Simple	116.0 ± 52.9	64.7 ± 33.9	1480.0 ± 588.7	6/0	0/6	0/6
	Exacerbated	202.6 ± 97.7	89.6 ± 100.0	1113.5 ± 683.4	8/0	1/7	1/7
	*P* value	0.071	0.014	0.16			

PDR: Proliferative diabetic retinopathy, PVR: Proliferative vitreoretinopathy.

**Table 5:** Cytokine levels in SOF.

Number of Samples		Simple PDR	Exacerbated PDR	Simple PVR	Exacerbated PVR
		10	7	6	8
FGF-2	(pg/mL)	27.8 ± 58.5	51.1 ± 48.3	17.8 ± 9.5	48.6 ± 34.6
IFNγ	(pg/mL)	0 ± 0	5.6 ± 14.9	0 ± 0	2.1 ± 3.8
IL-10	(pg/mL)	2.8 ± 3.9	12.5 ± 6.8	7.5 ± 8.1	15.7 ± 10.1
IL-12p40	(pg/mL)	2.1 ± 4.6	15.6 ± 9.8	7.2 ± 4.7	19.6 ± 22.0
IL-1β	(pg/mL)	1.4 ± 4.3	1.8 ± 4.7	0 ± 0	0 ± 0
IL-6	(pg/mL)	213.6 ± 423.5	467.0 ± 648.5	125.3 ± 72.6	676.3 ± 872.4
IL-8	(pg/mL)	44.8 ± 28.0	128.7 ± 139.1	88.2 ± 54.8	78.5 ± 29.9
MCP-1	(pg/mL)	5308.8 ± 2634.9	8615.7 ± 3204.1	8339.8 ± 3143.2	14820.0 ± 9231.6
TNFα	(pg/mL)	0.3 ± 1.0	1.8 ± 3.0	2.4 ± 3.5	0 ± 0
VEGF	(pg/mL)	52.1 ± 61.6	232.3 ± 275.3	87.4 ± 83.2	208.5 ± 275.3
TGFβ1	(pg/mL)	86.6 ± 99.9	224.2 ± 118.2	192.6 ± 135.3	225.3 ± 119.2

PDR: Proliferative diabetic retinopathy, PVR: Proliferative vitreoretinopathy.

### Differences in the cytokine levels between vitreous fluid and SOF

We next focused on the changes in the multiple cytokine levels from original vitreous fluid that were obtained at the primary vitrectomy surgeries to SOF. The average cytokine changes in each group are listed in Table 6. Although the numbers of samples were too small to be statistically analysed, when comparing the changes in each group, IL-12p40 levels were decreased only in the RRD, IL-6 levels were increased only in PDR, MCP-1 levels were largely reduced in RRD, TNFα levels were increased only in the MHRD, VEGF levels were largely reduced in simple PDR and TGFβ1 levels were increased only in MHRD.

**Table 6:** Changes in cytokine levels from the vitreous fluid to SOF.

Number of Samples		RRD	Simple PDR	Exacerbated PDR	MHRD
		4	3	4	2
FGF-2	(pg/mL)	14.0 ± 23.7	40.4 ± 135.1	14.9 ± 8.9	−145.8 ± 134.1
IFNγ	(pg/mL)	0 ± 0	0 ± 0	0 ± 0	0 ± 0
IL-10	(pg/mL)	−0.1 ± 0.6	−1.6 ± 2.7	2.9 ± 3.8	1.0 ± 1.1
IL-12p40	(pg/mL)	−1.2 ± 1.4	4.5 ± 7.8	3.4 ± 1.9	0.1 ± 0.2
IL-1β	(pg/mL)	0 ± 0	0 ± 0	0 ± 0	0 ± 0
IL-6	(pg/mL)	−41.3 ± 64.5	304.8 ± 921.0	270.1 ± 594.6	−5.6 ± 8.8
IL-8	(pg/mL)	−5.0 ± 22.2	−36.9 ± 61.5	17.9 ± 168.1	8.3 ± 11.5
MCP-1	(pg/mL)	−1713.5 ± 1541.9	388.7 ± 3655.0	−285.5 ± 1884.4	4.5 ± 529.6
TNFα	(pg/mL)	−0.1 ± 0.1	0 ± 0	−0.4 ± 3.1	2.6 ± 1.4
VEGF	(pg/mL)	−110.1 ± 85.1	−366.7 ± 600.4	54.6 ± 54.2	−6.7 ± 12.3
TGFβ1	(pg/mL)	−5.1 ± 35.9	−203.5 ± 54.9	−117.8 ± 193.1	50.2 ± 77.8

RRD: Rhegmatogenous retinal detachment, PDR: Proliferative diabetic retinopathy, MHRD: Macular hole-associated retinal detachment.

### SOF osmotic pressure

**Figure 4:**
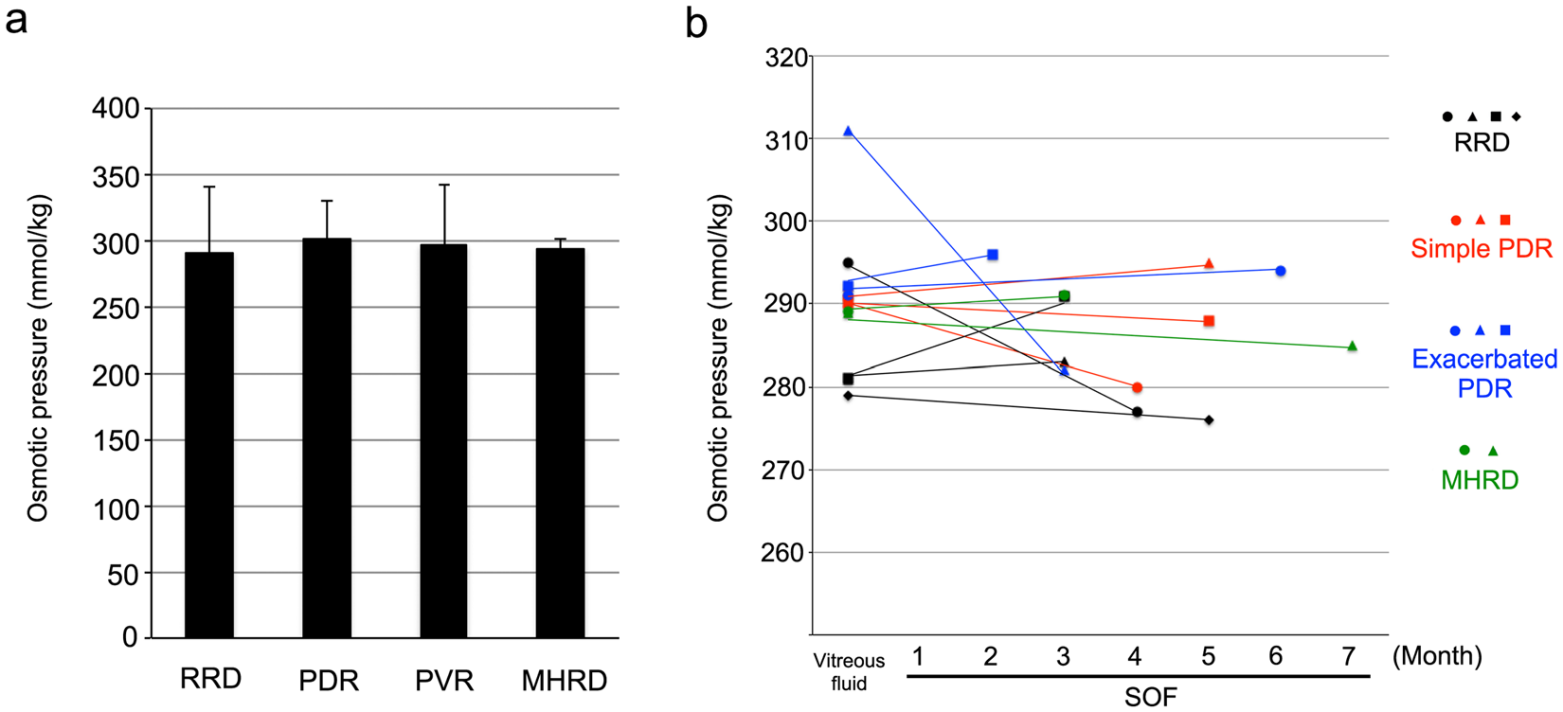
Changes in the osmotic pressure of vitreous fluid and sub-silicone oil fluid (SOF) from the same eyes. **(a)** The average SOF osmotic pressures from RRD, PVR, PVR, and MHRD. There were no significant difference between four groups. **(b)** The X-axis indicates the duration of SOF inside the eye and the Y-axis indicates the osmotic pressure. Note that the osmotic pressures in the vitreous fluid and SOF were not altered regardless of the disease or SOF duration. RRD: Rhegmatogenous retinal detachment, PDR: Proliferative diabetic retinopathy, MHRD: Macular hole-associated retinal detachment.

It is presumed that SOF is highly dense given the multiple cytokines present in a tiny amount of fluid. If so, it is possible that SOF has a high in osmotic pressure. Therefore, in this study, we measured the SOF osmotic pressure and compared it between groups. The SOF osmotic pressures were 291.9 ± 49.4, 301.9 ± 28.9, 297.0 ± 44.8 and 293.8 ± 7.6 mmol/kg in SOF with RRD, PDR, PVR and MHRD, respectively and were not significantly different according to the original diseases (*P* > 0.05, Fig. 4a). We also examined the changes in the osmotic pressures from original vitreous fluid to SOF from the same eyes. Of the 13 eyes from which both vitreous fluid and SOF were collected, osmotic pressures were measured in 12 (Fig. 4b). The average SOF osmotic pressure in all four groups was slightly decreased compared with the average vitreous fluid osmotic pressure. In addition, regardless of the duration of SOF in the eye, changes in osmotic pressure remained small (Table 7). These indicated that the osmotic pressures of SOF were not different depending on the different diseases and that differences in the osmotic pressures of SOF did not affect the clinical outcome.

**Table 7:** Osmotic pressures (mmol/kg) of the vitreous fluid and SOF from the same patients.

	Number of samples	Vitreous	SOF
RRD	4	284.0 ± 7.4	281.8 ± 6.9
Simple PDR	3	291.0 ± 1.0	287.7 ± 7.5
Exacerbated PDR	3	298.0 ± 11.3	290.7 ± 7.6
MHRD	2	289.0 ± 0.0	288.0 ± 4.2

RRD: Rhegmatogenous retinal detachment, PDR: Proliferative diabetic retinopathy, MHRD: Macular hole-associated retinal detachment.

## Discussion

In this study, we highlighted the importance of SOF in demonstrating the disease status in patients with refractory retinal diseases. Previous studies have indicated that SO influenced the pathogenesis of proliferative membrane growth. Lewis *et al*. and Zilis *et al*. reported that SO enhanced peri-silicone proliferation and formation of the preretinal membrane after using SO for advanced ﻿PVR^16, 17^ . Lambrou *et al*. performed an animal experiment to examine the effect of SO or SOF on retinal pigment epithelial (RPE) cells that are thought to be one of the major players in inducing PVR by biologically promoting epithelial-mesenchymal transition^18, 19^. They suggested that vitreous cavity SO had an increased mitogenic activity for RPE cells compared to gas-filled or fluid-filled-vitreous^19^. Asaria *et al*. examined TGFβ2, FGF-2, and IL-6 levels in SOF (their so-called retro-oil fluid) from 13 eyes with PVR but they did not collect SOF from eyes with PDR^15^. Nor did they measure VEGF, one of the most important cytokines in the current Ophthalmology both clinically and scientifically. Wickham *et al*. also reported on the biological relationship in intraocular SO between epiretinal membrane growth and macrophage infiltration. They precisely performed intraocular tissue immunostaining with markers for T- and B-lymphocytes, macrophages, and glial cells and could not conclude a direct relation between the duration of exposure to SO and the macrophage response^20^.

We demonstrated that IL-10, IL-12p40, IL-6, VEGF levels were higher in SOF from PVR than in SOF from RRD. Cytokines participate in the Th-1 or Th-2 types of immune response, which are different pathways of immunological reactions, orchestrated by T-helper cells. Cytokines such as IFN-γ and IL-12 are essential in the development of Th-1 immune responses, whereas IL-4, IL-10, and IL-12p40 are of major significance in Th-2 immune responses. IL-12 plays a certain role in the pathogenesis of idiopathic lung fibrosis (IPF) by regulating the Th-1 cell action^21^. Based on the biological studies suggesting that PVR and idiopathic lung fibrosis have many biological reactions in common^22, 23^, IL-12p40 possibly plays a certain role in the pathogenesis of PVR by regulating the T-helper cell action. IL-6 is one of the major pro-inflammatory cytokines, but it has also been reported to be a photoreceptor neuroprotectant in the experimental model of RD^24^. On the other hand, IL-10 is an anti-inflammatory cytokine, but it is upregulated by IL-6^25, 26^. We found significant increase in the IL-6 and IL-10 levels in SOF with PVR, although a previous study has demonstrated that IL-10 levels were not significantly higher in the vitreous of eyes with PDR compared with those in controls^27^. SOF in PVR contained higher MCP-1 levels than that in MHRD. Reportedly, MCP-1 levels in the vitreous fluid are correlated with IL-6 levels and the severity of PVR^28^. Interestingly, MCP-1 has been reported to play pivotal roles not only in RPE migration but also in photoreceptor apoptosis^29, 30^. Corroborating these results suggest that major pro-inflammatory cytokines, IL-6 and MCP-1, were extremely upregulated in SOF in PVR, and IL-10 was also reactively upregulated. When dividing two major proliferative retinal diseases into simple and exacerbated cases, SOF with exacerbated PDR but not exacerbated PVR demonstrated significantly higher FGF-2 and TGF-β1 levels than simple cases. FGF is related to wound-healing response in PVR, and TGF-β and FGF play crucial cooperative roles in fibrosis^31–33^. Our results suggested that FGF-2 and TGF-β1 accumulate greatly in SOF even in the simple cases of PVR.

Another notable result from this study was that osmotic pressure of the SOF did not significantly differ between the diseases nor did it differ from the osmotic pressure of from the original vitreous fluid obtained from the same eyes. For reference, human blood serum is approximately 290 mOsm/kg^34^. The osmotic pressures of the culture mediums that we often use for an *in vitro* assay, i.e., Dulbecco’s Modified Eagle’s Medium, Ham’s F-12, and RPMI-1640 are approximately 326–360, 282–312, and 264–292 mOsm/kg, respectively. Our study revealed that regardless of the duration of SO tamponade and original disease, osmotic SOF pressure was within the same range as human serum or culture medium, and the osmotic pressure of SOF is not relevant with the retinal toxicity.

There might be a question as to whether we should compensate for the cytokine concentration by the SOF volume. This idea might come from the fact that a larger SOF amount could possibly decrease the concentration of cytokines. However, our results (Table 3) indicated that there was no significant correlation between SOF volume and cytokine levels. Clinically the volume-based compensation of cytokines in SOF might not yield any additional important information. This is because it is the original concentration that contacts with the surface of the retina, regardless of the amount of SOF. Examining the direct relationship between the actual concentration of cytokines and clinical features of each disease is more important.

The limitation of this study was that we only had four SOF samples from MHRD, questioning the suitability of a statistical analysis. Compared to the other retinal diseases reviewed in this study, MHRD is relatively rare making it difficult to obtain a sufficient number of samples. However, biological information from SOF with MHRD is very important, primarily because of its long duration in the eye. Compared to the intraocular SO duration for other diseases, the SO and SOF duration were approximately 3 months longer for MHRD. Even after an average 7 month duration in the eye, most of the inflammatory cytokines in MHRD-associated SOF were very low. This indicated that the major factor that determines cytokine levels is not the duration of SOF but the original disease. Another important issue was the large SD in cytokine levels. Even after we collected >10 SOF samples from each of the major retinal diseases, SDs were still large. There might be several important factors that determine cytokine levels either inside the operated eye or the whole body. To overcome this problem, we collected both the vitreous fluid and SOF from the same eyes. We imagined that examining the changes in cytokine levels from the vitreous fluid to SOF in the same eye more accurately reflects the changes in the pathological condition in the treated eyes. Unfortunately, we could collect both the vitreous fluid and SOF from only small number of patients. The statistical analyses of the changes in cytokine levels from the vitreous fluid to SOF of larger number of samples will enable us to evaluate biological change more accurately.

Unfortunately, we could not perfectly understand why specific cytokines were high or low in specific diseases. For instance, it would be easy to assume that VEGF in the SOF from eyes with severe PDR would be higher than that for other diseases because the angiogenic importance of VEGF in many ocular diseases has been established. The same applies for a higher TGFβ expression in severe PVR. Therefore, examining the expression levels of these cytokines in SOF has a benefit of strongly predicting further clinical courses. On the other hand, little is known about the biological importance of some of the other cytokines such as IL-8 and IL-12p40 for ocular diseases. Further investigation is definitely required to understand these parameters. To promote the biological study of these cytokines, it is very important to establish easy methods to obtain SOF. In this study, we proposed a safe and easy method to extract SOF. Accumulating knowledge from SOF studies in multiple facilities will accelerate elucidating important biological information to predict clinical course after SO adjuvant application.

## Methods

### Sample collection and patient diseases

In this study, SOF and vitreous fluid were collected from the eyes of patients with RRD, PDR, PVR and MHRD. The group with PDR and PVR were then divided into two groups: simple PDR and simple PVR were classified as eyes in which SO evacuation surgeries were simple, without any additional procedures, and no unexpected medical complications such as re-proliferation of the fibrotic membrane or vitreous haemorrhage. Exacerbated PDR and exacerbated PVR were classified as eyes in which additional procedures were required, such as membrane removal, further revision vitrectomy surgery or disease recurrence before or after SO removal. The present study adhered to the guidelines of the Declaration of Helsinki and was approved by the Nagoya University Hospital Ethics Review Board. Written informed consent was obtained from all included patients.

### Extraction of SOF and vitreous fluid

All vitreous samples were collected by dry vitrectomy at the beginning of vitrectomy surgery using a vitrectomy cutter before infusion initiation^18^. All samples were immediately stored at −80 °C until use.

### Measurement of inflammatory cytokines

SOF and vitreous fluid were frozen and thawed only once before performing the MILLIPLEX MAP Human Cytokine/Chemokine Panel (Merck Millipore, Billerica, MA), a bead-based multiplex immunoassay which allows the simultaneous quantification of the following human cytokines: FGF-2, IFN-γ, IL-10, IL-12p40, IL-1β, IL-6, IL-8, MCP-1, TNFα and VEGF. Quantikine ELISA for human TGFβ1 (R&D Systems, Minneapolis, MN) was used to measure TGFβ1 following the manufacturer’s instruction. The values under detection sensitivity were defined as “0” in the statistical analyses.

### Measurement of osmotic pressure

A 10 mL sample of SOF or vitreous fluid was prepared for the osmotic measurement. The osmotic pressure of the sample was measured by a vapor pressure osmometer (Wescor VAPRO Model 5600, Wescor, Logan, UT, USA) following the manufacturer’s instructions.

### Statistics

Data were expressed as means ± standard deviation (SD; n = number of samples). In cases where one patient received treatment for both right and left eyes, each eye was counted individually (n = 2). Cytokine levels in multiple groups were analysed by the Kruskal-Wallis test and if significance was detected (*P* < 0.05), a Scheffe test was applied. Data from simple vs. exacerbated PDR and PVR were analysed by the Mann–Whitney U test. The correlation between the amount of SOF and cytokine levels were analysed by Spearman’s rank correlation. *P* values < 0.05 were considered statistically significant.

## Electronic supplementary material

Supplementary Video
